# Evaluating *Eucalyptus* leaf colonization by *Brasilonema octagenarum* (Cyanobacteria, Scytonemataceae) using *in planta* experiments and genomics

**DOI:** 10.7717/peerj.9158

**Published:** 2020-05-27

**Authors:** Danillo O. Alvarenga, Maione W. Franco, Kaarina Sivonen, Marli F. Fiore, Alessandro M. Varani

**Affiliations:** 1 Departamento de Tecnologia, Faculdade de Ciências Agrárias e Veterinárias, Universidade Estadual Paulista (UNESP), Jaboticabal, São Paulo, Brazil; 2 Department of Microbiology, Faculty of Agriculture and Forestry, University of Helsinki, Helsinki, Finland; 3 Departamento de Biologia Vegetal, Centro de Ciências Biológicas e da Saúde, Universidade Federal de Viçosa (UFV), Viçosa, Minas Gerais, Brazil; 4 Divisão de Produtividade Agroindustrial e Alimentos, Centro de Energia Nuclear na Agricultura, Universidade de São Paulo (USP), Piracicaba, São Paulo, Brazil

**Keywords:** Cyanobacteriota, Oxyphotobacteria, Phyllosphere, Lignocellulolytic enzymes, Virulence factors, Horizontal gene transfer

## Abstract

**Background:**

*Brasilonema* is a cyanobacterial genus found on the surface of mineral substrates and plants such as bromeliads, orchids and eucalyptus. *B. octagenarum* stands out among cyanobacteria due to causing damage to the leaves of its host in an interaction not yet observed in other cyanobacteria. Previous studies revealed that *B. octagenaum* UFV-E1 is capable of leading eucalyptus leaves to suffer internal tissue damage and necrosis by unknown mechanisms. This work aimed to investigate the effects of *B. octagenarum* UFV-E1 inoculation on *Eucalyptus urograndis* and to uncover molecular mechanisms potentially involved in leaf damage by these cyanobacteria using a comparative genomics approach.

**Results:**

Leaves from *E. urograndis* saplings were exposed for 30 days to *B. octagenarum* UFV-E1, which was followed by the characterization of its genome and its comparison with the genomes of four other *Brasilonema* strains isolated from phyllosphere and the surface of mineral substrates. While UFV-E1 inoculation caused an increase in root and stem dry mass of the host plants, the sites colonized by cyanobacteria on leaves presented a significant decrease in pigmentation, showing that the cyanobacterial mats have an effect on leaf cell structure. Genomic analyses revealed that all evaluated *Brasilonema* genomes harbored genes encoding molecules possibly involved in plant-pathogen interactions, such as hydrolases targeting plant cell walls and proteins similar to known virulence factors from plant pathogens. However, sequences related to the type III secretory system and effectors were not detected, suggesting that, even if any virulence factors could be expressed in contact with their hosts, they would not have the structural means to actively reach plant cytoplasm.

**Conclusions:**

Leaf damage by this species is likely related to the blockage of access to sunlight by the efficient growth of cyanobacterial mats on the phyllosphere, which may hinder the photosynthetic machinery and prevent access to some essential molecules. These results reveal that the presence of cyanobacteria on leaf surfaces is not as universally beneficial as previously thought, since they may not merely provide the products of nitrogen fixation to their hosts in exchange for physical support, but in some cases also hinder regular leaf physiology leading to tissue damage.

## Introduction

Cyanobacteria are microorganisms that colonize a wide variety of habitats, including the phyllosphere, either as free-living colonies or in symbiotic relationships. Cyanobacterial symbioses with eukaryotes are usually mutualistic, with cyanobionts providing partners with fixed carbon or nitrogen and/or defending them with toxins or sunscreens in exchange for protection from extreme environmental conditions and predation (Usher, Bergman & Raven, 2007; Adams et al., 2013; Rikkinen, 2007). *Brasilonema octagenarum* differs from other symbiotic cyanobacteria in this regard by presenting strains that are capable of damaging plant leaves (Aguiar et al., 2008), a behavior that as far as is currently known is unique in this phylum.

Thus far, *Brasilonema* strains have been found on subaerophytic habitats from tropical and subtropical environments of Brazil, French Antilles, Mexico, and USA with ten species described, namely *B. bromeliae*, *B. angustatum*, *B. burkei*, *B. geniculatum*, *B. lichenoides*, *B. octagenarum*, *B. roberti-lamii*, *B. sennae*, *B. terrestre*, and *B. tolantongensis* (Fiore et al., 2007; Aguiar et al., 2008; Sant’Anna et al., 2011; Vaccarino & Johansen, 2012; Becerra-Absalón et al., 2013; Rodarte et al., 2014; Miscoe et al., 2016; Villanueva et al., 2018; Villanueva et al., 2019). The distribution of the genus *Brasilonema* is most likely pantropical (Hauer, 2010; Kaštovský et al., 2010), thus new species are likely to be found in previously unsurveyed geographical regions and habitats. Nevertheless, although other strains have been isolated from the phyllospheres of different host species, plant damage has so far only been observed in *B. octagenarum*.

**Figure 1:**
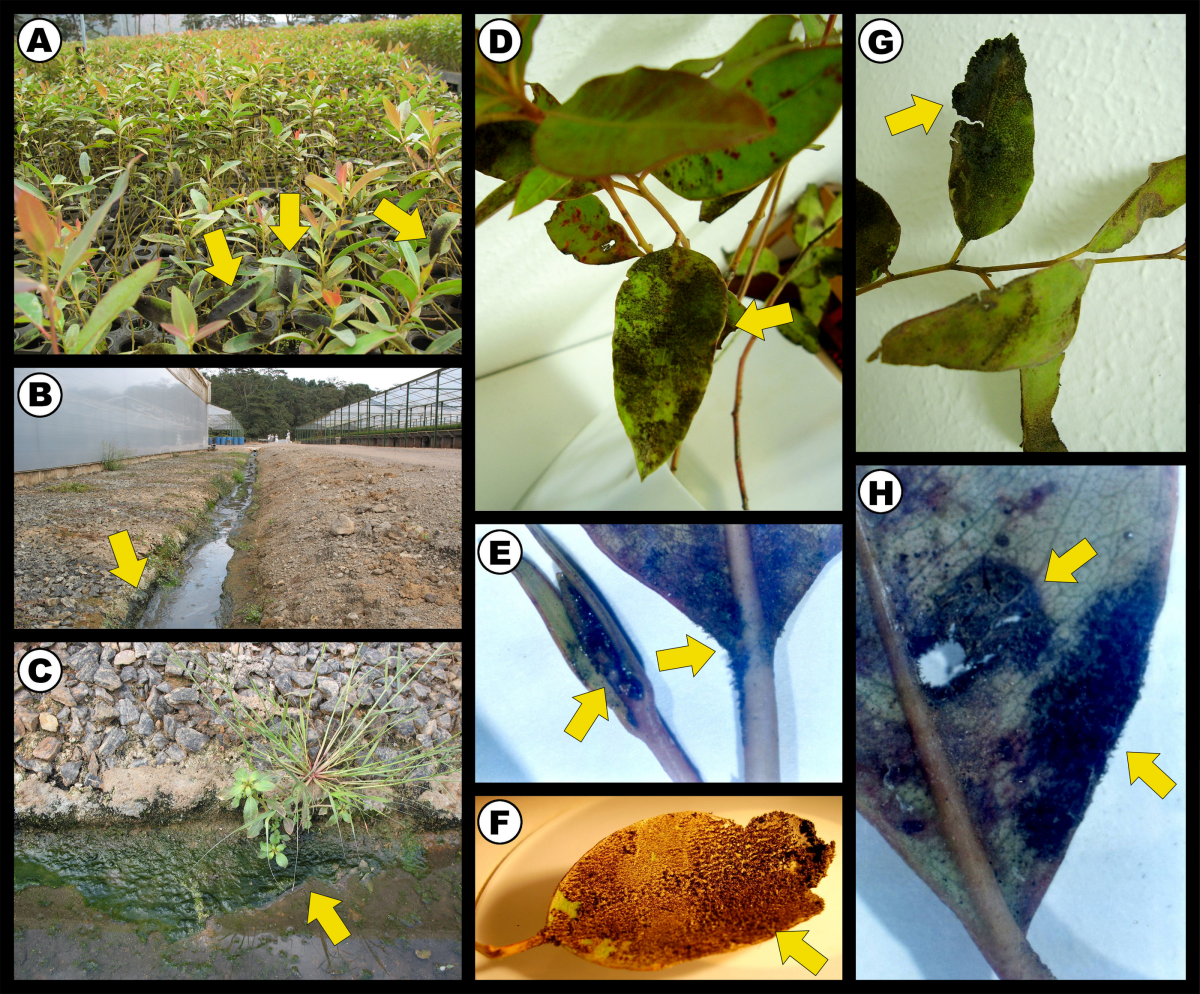
Infection of *Eucalyptus urograndis* saplings by *Brasilonema octagenarum* under greenhouse conditions in Minas Gerais, southeast Brazil. Arrows point to cyanobacterial mats. (A) *E. urograndis* nurseries presenting visible growth of *B. octagenarum* mats on leaf surfaces. (B–C) *B. octagenarum* biofilms colonizing mineral substrates in contact with water drained from a greenhouse containing *E. urograndis* plants colonized by *B. octagenarum* UFV-E1. (D–H) Limbs, petiole, and apical buds of young *E. urograndis* leaves colonized by* B. octagenarum* UFV-E1 presenting pathogenic symptoms, including necrosis. Photo credit: Maione W. Franco.

Ultrastructural analyses have previously shown that*B. octagenarum* UFV-E1 mats can invade the mesophylls of *Eucalyptus* leaves under nursery conditions, which results in internal cell damage and necrosis and leads to reduced rates of photosynthesis and stomatal gas exchange, eventually causing reduction in growth and harming the productivity of the host plant (Aguiar et al., 2008). This cyanobacterium colonized the aerial parts of *Eucalyptus* saplings and mineral substrates at the vicinity of a greenhouse (Figs. 1A–1C), with leaves colonized by strain UFV-E1 showing damage to limbs, petiole, and apical buds (Figs. 1D–1H). The molecular mechanisms by which this interaction occurs have not been elucidated yet, and whether the cellular damage is caused directly by the cyanobacterium or indirectly by its epiphytic growth is also currently unknown.

Since similar leaf damage has not been previously observed in cyanobacteria-plant interactions, it is possible that *Brasilonema octagenarum* has acquired the capacity of infecting plants from phytopathogenic organisms by horizontal gene transfer. Therefore, this work aimed to experimentally reproduce under controlled conditions the effects of *B. octagenarum* UFV-E1 colonization on eucalyptus leaves; to characterize its genome to search for molecular mechanisms potentially involved in leaf damage by this strain; and to evaluate whether horizontal gene transfer had any role on the evolution of these mechanisms. For these purposes, the aerial parts of *Eucalyptus urograndis* saplings were inoculated with *B. octagenarum* UFV-E1 cultures and subsequent changes in morphology, physiology and growth of the plants were evaluated. Afterwards, the genomes of *B. octagenarum* UFV-E1 and four other *Brasilonema* spp. strains were sequenced, characterized and compared and the presence of sequences orthologous to proteins previously reported as involved in plant-pathogen interactions was inquired.

## Material and Methods

### Leaf colonization

*Brasilonema octagenarum* was originally found and isolated by Aguiar et al. (2008) in an eucalypt nursery in Minas Gerais, southeastern region of Brazil. *B. octagenarum* UFV-E1 was grown in liquid BG-11 medium (Allen, 1968) at temperatures of 25 ± 2 °C, photoperiod of 16/8 h light/dark and irradiance of 115 µm photons m^−2^s^−1^. After 30 days, the cultures were filtered using general purpose filter paper under sterile conditions and concentrated into 10% of the initial volume of 3 L to eliminate supernatants and facilitate the inoculation of leaves. Six *Eucalyptus urograndis* clonal plants were transplanted to 2 L pots containing substrate and acclimatized for two weeks in a greenhouse under the controlled conditions of 25−30 °C temperature and above 80% relative humidity. Cell suspensions were inoculated homogeneously on the aerial parts of each plant, except for six uninoculated plants used as controls. After 30 days, the cyanobacterial colonies were mechanically removed from leaf limbs using gentle fingertip movements and the middle portion of the leaves was used for extraction and quantification of pigment content. Three discs were taken from different, fresh leaves (Ø  = 0.59 cm) in each control or inoculated plant and immersed in 7 mL of dimethylsulfoxide (DMSO) (Wellburn, 1994) in the dark for 24 h. Absorbance was determined at *λ* of 665.1, 680 and 450 for chlorophyll *a*, chlorophyll *b* and carotenoids, respectively. Stem height and leaf area were evaluated, and stem, root and leaf dry masses were measured after oven dehydration at 75 °C for 72 h. The leaf area was measured using a leaf area integrator (Delta-T Devices, Cambridge, UK). The averages of the data obtained were compared with the Tukey test (significance level *P* < 0.05) using SAS 9.2 (SAS Institute, Cary, USA).

### Anatomical evaluation

Leaf blade samples collected from inoculated and control plants were fixed with 50% FAA solution (formaldehyde, acetic acid and 70% ethanol) for 48 h and stored in 70% ethanol (Johansen, 1940). After the middle blade region was sectioned and the samples were embedded in Histosec (Merck, Kenilworth, USA), 10 µm cross sections were obtained with an automatic advance rotary microtome. Part of the cuts were stained with safranin/astra blue (Bukatsch, 1972) for observing the general structure and presence of phenolic compounds, and the other part was stained with scarlet Sudan (Sass, 1951) for cuticle observation. Next, the cuts were mounted on Permount synthetic resin (Thermo Fisher Scientific, Waltham, USA). Sections were examined and images were captured with the Olympus AX-70 photomicroscope system (Olympus, Shinjuku, Japan) using the Image-Pro Discovery software (MediaCybernetics, Silver Spring, USA).

### High-throughput sequencing

*Brasilonema octagenarum* UFV-E1, *B*. *octagenarum* CENA114, *B. octagenarum* UFV-OR1, *B. bromeliae* SPC 951 and *Brasilonema* sp. UFV-L1 were grown as non-axenic, unicyanobacterial cultures in 125 mL Erlenmeyer flasks containing 50 mL of sterile BG-11_0_ liquid medium (Allen, 1968; Stanier et al., 1971). For the reduction of associated microbes, the samples were washed with ultrapure water, 0.05% Extran, 0.85% NaCl, and a solution composed of 50 mM NaCl, pH 7.5 10 mM Tris-HCl and pH 8.0 2.5 mM EDTA. After inoculation, incubation was carried out at 25 ± 1 °C with a photoperiod of 14 h of light and 10 h of darkness under fluorescent light of 40 µmol photons m^−2^ s^−1^. Two-week-old cultures were centrifuged for 10 min at 7, 690 × g and cells were collected for DNA extraction using a modified version of the protocol established by Lin et al. (2010). Paired-ends libraries were produced for all strains with the Nextera XT DNA Sample Prep Kit (Illumina) according to manufacturer’s instructions. Additionally, mate-paired libraries were prepared from 8 kbp inserts for strains UFV-E1 and CENA114 using the Nextera Mate Pair Library Prep Kit (Illumina) according to the manufacturer’s protocols in order to improve assemblies and obtain complete genomes for the main strain of this study and the strain not isolated from phyllosphere. Sequencing was carried out in the HiSeq 2500 platform using the HiSeq v4 Reagent Kit (Illumina) following the instructions provided by the manufacturer.

### Genome assembly

Bases with qualities lower than Phred 28, adapters and sequences shorter than 50 bp were removed from the datasets with Trimmomatic 0.36 (Bolger, Lohse & Usadel, 2014). Mate-paired libraries were further refined with NxTrim 0.4.2 (O’Connell et al., 2015) for the separation of true mate-paired reads. *De novo* assemblies were carried out using SPAdes 3.11.1 (Bankevich et al., 2012) and MaSuRCA 3.2.4 (Zimin et al., 2013). Kraken 1.0 (Wood & Salzberg, 2014) and MetaBAT 2.12.1 (Kang et al., 2015) were used for identifying cyanobacterial sequences among the assembled contigs. Platanus 1.2.4 (Kajitani et al., 2014) was used for further scaffolding and gap closing. Genome completeness and contamination were estimated with CheckM 1.0.7 (Parks et al., 2014).

### Genome annotation

The assembled genomes were automatically annotated with the NCBI Prokaryotic Genome Annotation Pipeline (Tatusova et al., 2016). When necessary, manual curation of annotations was performed with Artemis 16.0.17 (Carver et al., 2012) and BLAST 2.6.0+ (Camacho et al., 2009). Functional identification of translated protein sequences into orthologous groups was performed with eggNOG 4.5.1 (Huerta-Cepas et al., 2016), Blast2GO 5.2 (Götz et al., 2008) and WEGO (Ye et al., 2018). The RAST server (Overbeek et al., 2014; Brettin et al., 2015) was used for predicting the subsystems present in the assembled genomes. Protein sequences from the Pathogen-Host Interactions Database version 4.5 (Urban et al., 2020) were used to uncover genes encoding proteins similar to known virulence factors by using TBLASTN with cut-off values of 90% for coverage and 60% for positive-scoring amino acid matches. Translated protein sequences for hypothetical proteins were retrieved from the assembled genomes and their domains were predicted with the Phobius web server (Käll, Krogh & Sonnhammer, 2007). Predictions for possible effectors from the type III secretion system were performed with the EffectiveDB web server (Eichinger et al., 2016).

### Prediction of mobile genetic elements

The occurrence of mobile genetic elements in the genomes obtained was verified according to the protocols described in Alvarenga et al. (2018). Insertion sequences were predicted with OASIS 8∕11∕08 (Robinson, Lee & Marx, 2012), ISEScan 1.5.4.3 (Xie & Tang, 2017) and the ISfinder database (Siguier et al., 2006; Siguier et al., 2012). Prophages were predicted with PhiSpy 3.2 (Akhter, Aziz & Edwards, 2012) and VirSorter 1.0.3 (Roux, Enault & Hurwitz, 2015). CRISPRs were detected with MinCED 0.2.0 (https://github.com/ctSkennerton/minced). For the uncovering of genomic regions with anomalous content, Alien_Hunter 1.7 (Vernikos & Parkhill, 2006), OligoWords 1.2.1 and SeqWord Sniffer 2.0 (Ganesan et al., 2008) were used.

### Genome comparisons and phylogenomic analyses

OrthoANIu 1.2 (Yoon et al., 2017) and USEARCH 10.0.240 (Edgar, 2010) were used for estimating average nucleotide identity among the assembled genomes. Orthologous protein clusters shared among the strains were surveyed by the OrthoVenn server (Wang et al., 2015). The assembled genomes were included in a dataset with 154 complete and nearly complete cyanobacterial genomes retrieved from the NCBI RefSeq database (O’Leary et al., 2016) for selection of marker sequences with Metaxa 1.0.2 (Bengtsson et al., 2011) and Phyla-AMPHORA 03∕19∕13 (Wang & Wu, 2013). Multiple sequence alignments for sixty single-copy translated protein sequences shared by all genomes and 16S rRNA gene sequences were produced with MAFFT 7.309 (Katoh & Standley, 2013) and concatenated into a single alignment. A maximum likelihood phylogenomic tree was reconstructed from the concatenated translated proteins/16S rRNA gene alignment with RAxML 8.2.9 (Stamatakis, 2014) using the best-fit evolutionary models for each partition as calculated with the phylogenomics-tools pipeline (https://github.com/kbseah/phylogenomics-tools). The tree was visualized with FigTree 1.4.2 (http://tree.bio.ed.ac.uk/software/figtree/) and edited with Inkscape 0.92 (https://inkscape.org/).

## Results

### *Eucalyptus* leaf inoculation

We first reinoculated *B. octagenarum* strain UFV-E1 on *E. urograndis* leaves to check if the cyanobacterial colonization and its effects previously found under natural conditions could be artificially replicated on seedlings*.* One month after inoculation, *E. urograndis* leaves colonized by strain UFV-E1 showed a significant decrease of 19% in the Chl *a*/Chl *b* ratio in the areas colonized by the cyanobacterium (Table 1). When the content of the pigments Chl *a*, Chl *b* and carotenes were analyzed individually, significant alteration in the colonized areas in relation to the control samples was not verified (Table 1). The inoculated plants had an increase in root and stem dry mass (19% and 23%, respectively) and stem height (7%), while leaf dry mass and area showed no significant differences between inoculated plants and controls (Table 2).

**Figure 2:**
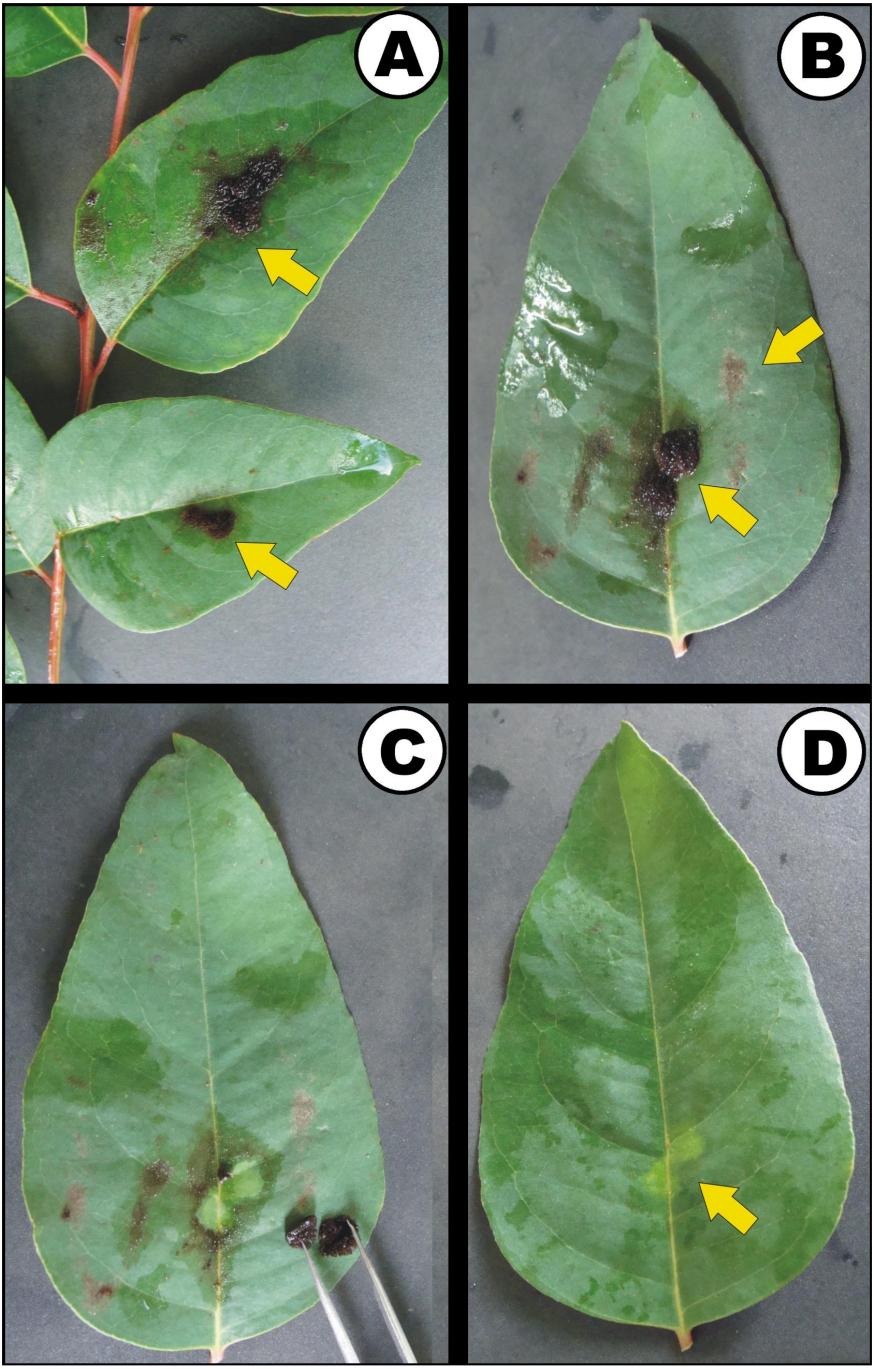
*Eucalyptus urograndis* leaves colonized by *Brasilonema octagenarum* UFV-E1. (A) General appearance of colonized leaves presenting conspicuous growth of cyanobacterial filaments. (B) Cyanobacterial mats covering the central vein of the host leaf. (C) Removal of cyanobacterial colonies from the leaf surface. (D) Discoloration in leaf areas previously covered by cyanobacterial colonies. Photo credit: Maione W. Franco.

**Figure 3:**
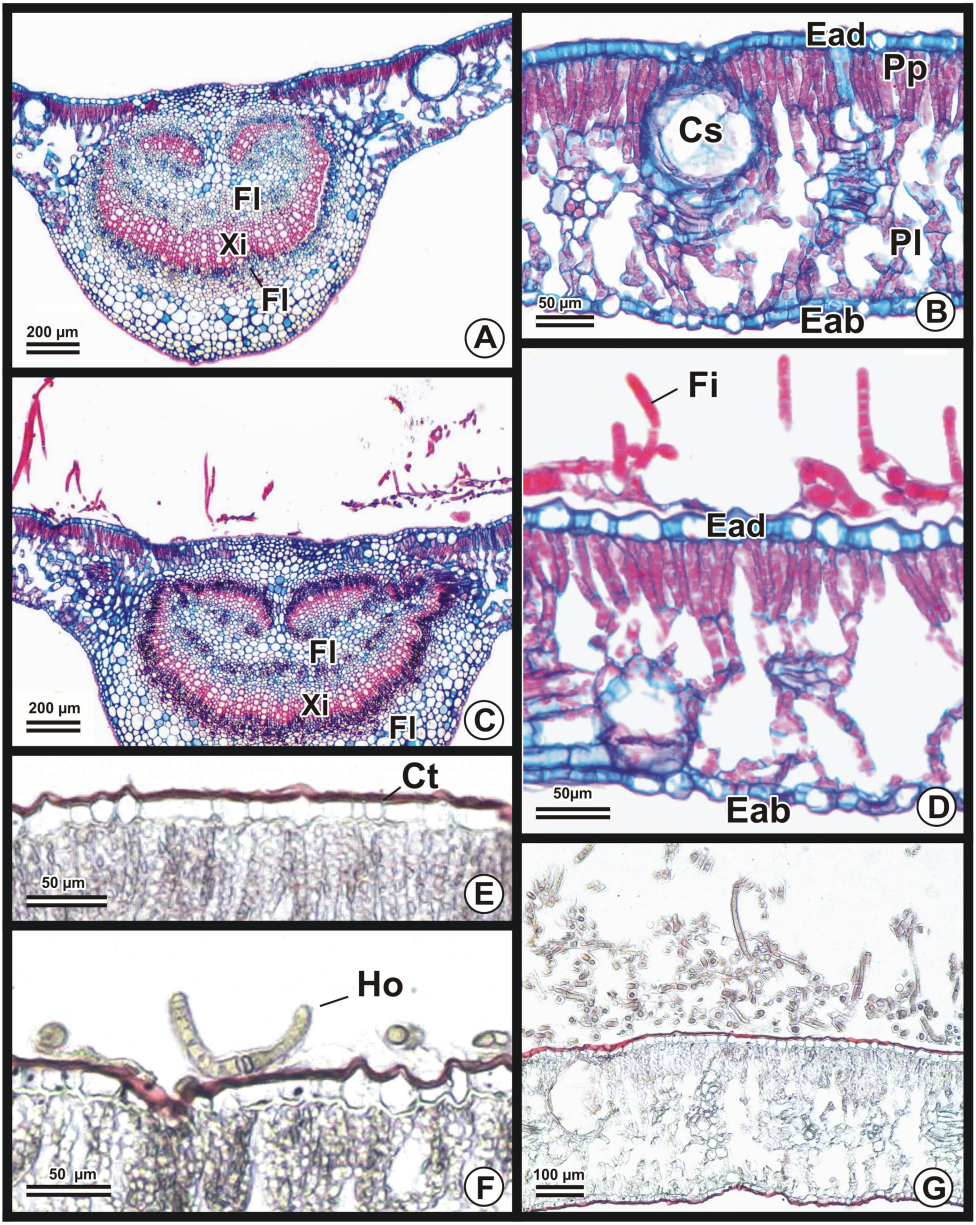
Photomicrographs of cross sections from *Eucalyptus urograndis* leaves stained with safranin/astra blue (A–D) or Sudan (E–G). (A) Cross section of a control leaf, showing the xylem (Xy) and phloem (Fl) in a general view of its central rib. (B) Portion of a control leaf blade in higher magnitude, showing more details of the epidermis of the adaxial (Ead) and abaxial (Eab) surfaces, a secretory cavity (Cs), the palisade parenchyma (Pp) and the lacunar parenchyma (Pl). (C) Central rib colonized by *Brasilonema octagenarum*, with filaments on the adaxial side of the epidermis. (D) Portion of the leaf lamina evidencing the fasciculate growth pattern of the cyanobacterial filaments (Fi) on the adaxial surface. (E) Control leaf epidermis showing a red-orange cuticle (Ct). (F) Integral cuticle in inoculated plants, evidencing an hormogonium (Ho). (G) Portion of the colonized leaf blade, presenting a dense layer of filaments of *B. octagenarum* on the adaxial side with an intact cuticle. Photo credit: Maione W. Franco.

*B. octagenarum* UFV-E1 colonized *E. urograndis* blades at points scattered along leaf surfaces (Figs. 2A and 2B). After the removal of the cyanobacterial colonies (Fig. 2C), the regions previously covered by denser colonies showed alterations in pigmentation (Fig. 2D). Transverse sections of *E. urograndis* leaves from the control group showed a prominent central rib on the abaxial face, presenting a bicollateral vascular bundle on both sides and fundamental parenchyma (Fig. 3A). The epidermis was unstratified, covered by cuticle, showing stomata mainly on the abaxial face. The mesophyll was dorsinventral, composed of palisade parenchyma formed by one or two layers of cells, and lacunar parenchyma, formed by three or four layers (Fig. 3B). In the plants colonized by *B. octagenarum*, colonies were observed mainly on the adaxial face (Figs. 3C and 3D). No structural changes were observed caused by colonization (Fig. 3C) in the regions of the central rib or the remainder of the leaf lamina (Fig. 3D). The cuticle remained without changes in the regions colonized by *B. octagenarum* (Figs. 3F and 3G) in comparison with the controls (Figs. 3E and 3G).

**Table 1:** Pigment content in leaf areas of *Eucalyptus urograndis* plants colonized by *Brasilonema octagenarum*. *Significant effect at *P* ≤ 0.05, Tukey test.

**Sample**	**Chl*a*(µg cm**^−2^**)**	**Chl*b*(µg cm**^−2^**)**	**Carotenoids (µg cm**^−2^**)**	Chl*a*/b*
controls	15.64 ± 0.47	6.78 ± 0.29	2.98 ± 0.21	2.32 ± 0.10
inoculated plants	14.45 ± 0.44	7.42 ± 0.33	2.93 ± 0.30	1.95 ± 0.06

**Table 2:** Growth variables evaluated in *E. urograndis* plants after inoculation with *B. octagenarum*. Significant effects at * *P* ≤ 0.05 and ** *P* ≤ 0.01, Tukey test.

**Sample**	**Root dry weight (g)**	**Stem dry weight (g)**	**Leaves dry weight (g)**	**Leaf area (cm**^**2**^**)**	**Stem height (cm)**
controls	15.11* ± 0.63	23.07** ± 1.01	21.22 ± 1.82	3,586.5 ± 255.3	83.3** ± 1.3
inoculated pants	17.94 ± 0.73	28.36 ± 0.99	22.47 ± 0.49	4,136.5 ± 147.5	89.5 ± 1.2

### Genomic sequencing and characterization

**Figure 4:**
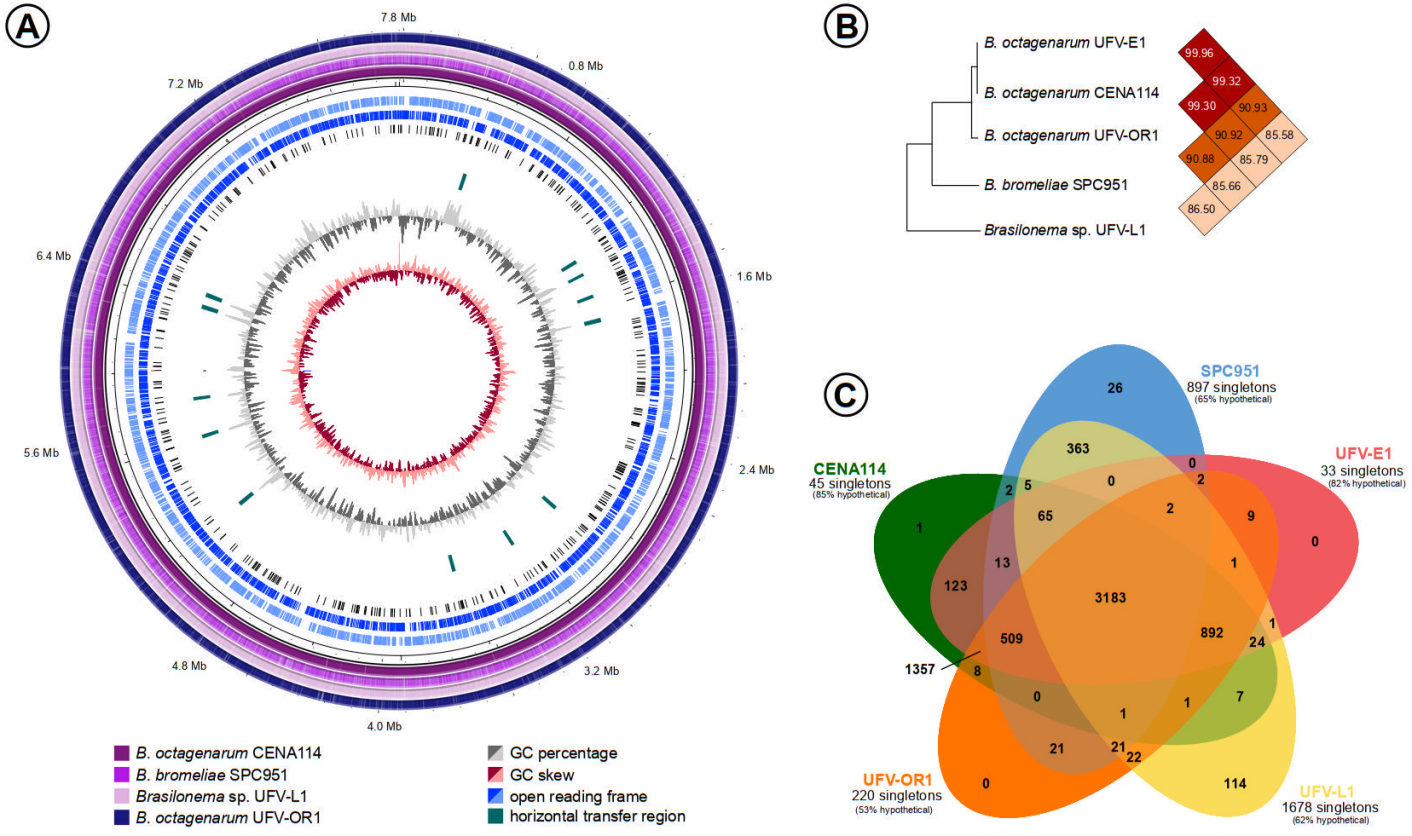
Comparisons between the *Brasilonema* genomes characterized in this work. (A) Circular map of the nearly complete genome of *B. octagenarum* UFV-E1 compared to the other *Brasilonema* genomes indicating regions with possible origin by horizontal transfer. (B) Percentages of average nucleotide identities shared between the *Brasilonema* genomes obtained. (C) Quantification of protein clusters orthologous between *Brasilonema* strains and estimation of the amount of singletons encoded in the analyzed genomes.

The genome sequencing of *Brasilonema* strains resulted in the assembly of nearly complete genomes for *B. octagenarum* UFV-E1 and *B. octagenarum* CENA114 while draft genomes were obtained for *B. octagenarum* UFV-OR1, *B. bromeliae* SPC 951 and *Brasilonema* sp. UFV-L1 (Fig. 4A, Table 3). The genome sequences obtained in this work were deposited in the NCBI Genome database under the accession numbers CP030118 –CP030123, QMEA00000000, QMEB00000000, QMEC00000000. Predictions for the nearly complete chromosomes of strains UFV-E1 and CENA114 uncovered 17 and 14 genome islands, respectively. Additionally, 158 insertion sequences were found in both sequences. No prophages were detected in those chromosomes. On the other hand, two remnant prophage regions were predicted in each plasmid from both strains. Except for the UFV-E1 190 kbp plasmid, two genome islands were also detected in those plasmids. Moreover, 11 additional insertion sequences were found in the 250 kbp plasmids, while 18 and 16 insertion sequences were detected in the 190 kbp plasmids from UFV-E1 and CENA114, respectively. Finally, 9 CRISPR loci were predicted in both nearly complete chromosomes. Average nucleotide identity (ANI) among strains UFV-E1, CENA114, and UFV-OR1 was calculated as 99%, showing that they are actually representatives of the same species, *B. octagenarum*, which presented 90 and 85 ANI percentages when their genomes were compared to the genomes of *B. bromeliae* SPC 951 and *Brasilonema* sp. UFV-L1, respectively (Fig. 4B). Clustering analyses revealed a large number of orthologous protein clusters shared among the analyzed *Brasilonema* genomes, with a very small amount of protein clusters exclusive to each strain (Fig. 4C).

**Table 3:** Genomic features of the *Brasilonema* spp. strains characterized in this work.

**Feature**	**UFV-E1**	**CENA114**	**UFV-OR1**	**SPC 951**	**UFV-L1**
completeness (%)	99.52	100.00	99.28	90.48	99.40
contamination (%)	2.89	2.17	3.01	2.37	1.20
total size (bp)	8,258,380	8,226,398	8,094,337	6,277,767	8,321,598
–chromosome	7,816,330	7,782,641	—	—	—
–plasmid 1	251,877	252,036	—	—	—
–plasmid 2	190,173	191,721	—	—	—
coding sequences (bp)	6,559,505	6,529,766	6,271,238	4,742,865	6,443,634
–chromosome	6,198,811	6,170,908	—	—	—
–plasmid 1	204,544	204,193	—	—	—
–plasmid 2	156,150	154,665	—	—	—
GC content (%)	42.49	42.50	42.48	42.97	41.97
genes	6,950	6,929	6,908	5,693	7,214
protein-coding genes	6,395	6,371	6,309	5,207	6,652
pseudogenes	497	502	541	432	516
RNA genes	58	56	58	54	46
ncRNA	4	4	4	4	4
genes with assigned subsystems	1,394	1,388	1,427	1,137	1,397
genes assigned to COG categories with known function	3,440	3,426	3,379	2,741	3,389
hypothetical proteins	2,081	2,092	2,074	1,695	2,314

**Figure 5:**
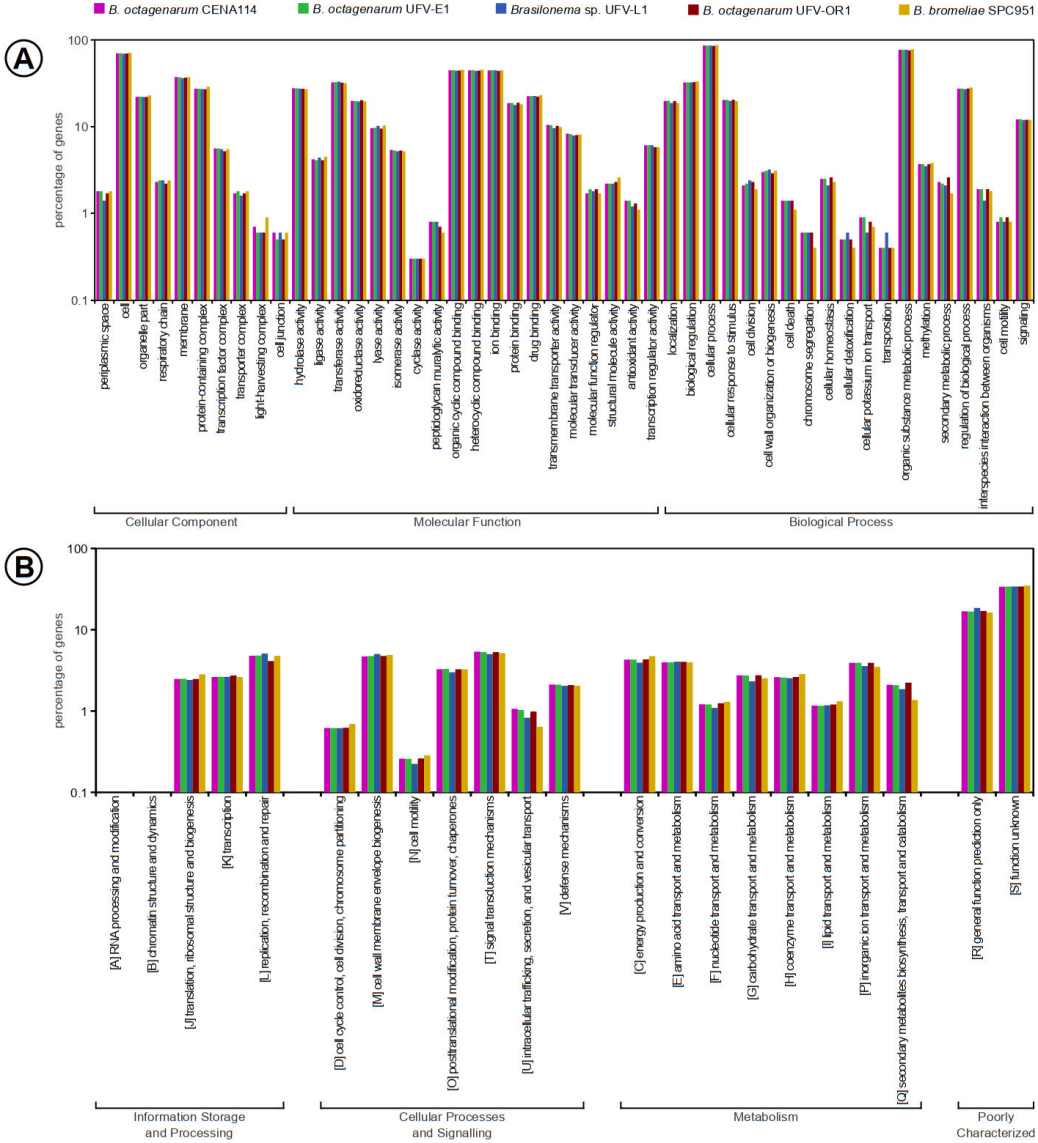
Functional annotations of genes included in the *Brasilonema* spp. genomes characterized in this work, evidencing high similarity between strains and species in this genus. (A) Gene Ontology terms associated with the predicted properties of the products encoded in *Brasilonema* genomes. (B) Distribution of COG categories among *Brasilonema* genomes, showing that they encode a large number of poorly characterized proteins. COG categories W, Y, and Z did not present any matches and thus were suppressed.

In the virulence, disease and defense subsystem predicted in the nearly complete *B. octagenarum* genomes, genes involved in resistance to fluoroquinolone- and betalactam-based antibiotics were found, in addition to genes for resistance to copper, mercury, arsenic, chromium and cobalt/zinc/cadmium, suggesting that these cyanobacteria may have some tolerance to environmental disturbances, such as contamination by toxic element. However, subsystem coverage was very low in the assembled genomes, as merely 18% of sequences in the *B. octagenarum* genomes could be included into known subsystems, while subsystem coverage in the *B. bromeliae* SPC 951 and *Brasilonema* sp. UFV-L1 genomes amounted to 17% of their content. Similarly, the most abundant functional categories for clusters of orthologous groups (COGs) in all genomes were those with general function prediction only or unknown function. Overall all strains presented very similar number of genes related to known COG and GO terms (Fig. 5, Table S2), suggesting that these species may perform similar ecological roles. The analysis of translated protein sequences from genes annotated as encoding hypothetical proteins with the Phobius webserver revealed that roughly three quarters are likely non-cytoplasmic and approximately one quarter of these sequences present transmembrane domains (Table 4). This suggests that a considerable number of yet uncharacterized proteins are exported from the cells, with some of them possibly involved in ecological interactions.

**Figure 6:**
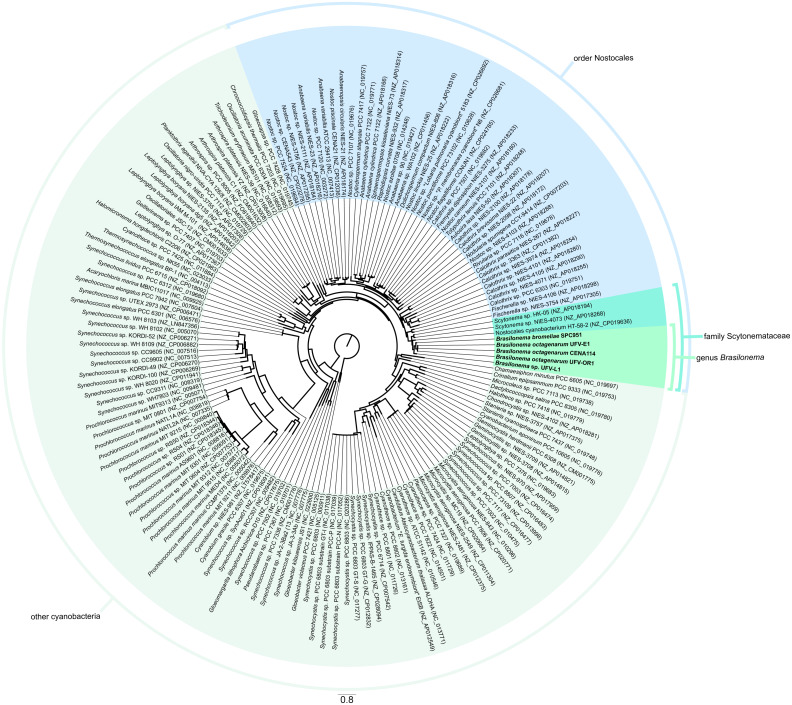
Phylogenomic tree reconstructed by maximum likelihood. Analysis based on alignments of sequences of the 16S rRNA gene and sixty proteins codified in single-copy genes present in the genomes from the strains evaluated in the present work (highlighted in bold) and nearly complete cyanobacterial genomes and chromosome scaffolds from the NCBI RefSeq database.

In accordance with the latest system of cyanobacterial classification by Komárek et al. (2014), the phylogenomic analysis showed that the *Brasilonema* strains were closely related to *Scytonema* representatives, thus confirming previous polyphasic descriptions of the family Scytonemataceae, which in turn clustered inside the order Nostocales (Fig. 6), as expected based on previous works on the taxonomic positioning of these cyanobacteria (Fiore et al., 2007; Komárek et al., 2014).

### Evaluation of sequences that might be involved in plant interactions

Twenty five secondary metabolite gene clusters were predicted in the nearly complete genomes of strains UFV-E1 and CENA114, including clusters encoding the pathways for polyketides, non-ribosomal peptides, post-translationally-modified peptides, bacteriocins, terpenes, indoles, and phenazines, most of which presented low similarity to reference clusters involved in the biosynthesis of known molecules. Five gene clusters, however, presented 100% similarity to references for clusters that encode the biosynthesis of the cyclic non-ribosomal peptides anabaenopeptin and nostopeptolide, the sesquiterpene geosmin, the polyketide 1-heptadecene, and the UV-protecting amino acid shinorine. Similarity to references for the biosynthesis of known cyanotoxins, which might be involved in plant-pathogen interactions, was not found in any of the assembled genomes.

Annotations in all the *Brasilonema* genomes uncovered several genes encoding enzymes that likely have cellulose, lignin and xylan as substrates (Table 5), which may therefore have a role on leaf colonization and interact with host cell walls. Other enzymes that might have some role during plant colonization have also been annotated in the genomes of all the *Brasilonema* strains, including proteins involved in chitin and protein degradation, auxin biosynthesis and biofilm formation through exopolysaccharide biosynthesis (Table 6).

Protein sequences with similarity to relevant virulence factors from plant pathogens deposited in the Pathogen Host Interaction database were found encoded in the genomes of all five *Brasilonema* strains analyzed in the present work (Table 7). Similarities were found with proteins from phytopathogenic bacteria (*Burkholderia glumae*, *Erwinia amylovora*, *Pantoea ananatis*, *Ralstonia solanacearum*, *Xanthomonas campestris*, *X. citri* and *X. oryzae*), fungi (*Botrytis cinerea*, *Colletotrichum lagenaria, Fusarium graminearum, F. oxysporum, Magnaporthe oryzae* and *Parastagonospora nodorum*) and nematodes (*Bursaphelenchus xylophilus*), which target leaves, shoots, roots, cones, panicles, seedlings, cotyledons or fruits. Among the set of virulence factors with homologous sequences in the *Brasilonema* genomes (36 proteins), the majority (26 proteins) can be considered essential to phytopathogenicity since the virulence of the organisms that express these factors may be reduced or even lost after deleterious mutations occur in their coding sequences (Table 7).

**Table 4:** Domains predicted from hypothetical protein sequences encoded in *Brasilonema* genomes.

Strain	Cytoplasmic	Non cytoplasmic	Non cytoplasmic + signal peptide	Cytoplasmic + non cytoplasmic + transmembrane	Cytoplasmic + non cytoplasmic + transmembrane + signal peptide
*B. octagenarum* UFV-E1	98	1,200	324	417	42
*B. octagenarum* CENA114	96	1,206	329	415	44
*B. octagenarum* UFV-OR1	99	1,195	313	399	42
*B. bromeliae* SPC 951	83	1,019	204	316	44
*Brasilonema* sp. UFV-L1	140	1,410	278	416	50

**Table 5:** Annotations for enzymes encoded in *Brasilonema* genomes potentially having plant polysaccharides as substrates.

**Protein**	**Substrate**	**UFV-E1**	**CENA114**	**UFV-OR1**	**SPC 951**	**UFV-L1**
cellulase M	cellulose	+	+	+	+	+
1,4-beta-glucanase / beta-1,4-glucanase	cellulose	+	+	+	+	+
endo-1,4-beta-glucanase	cellulose	+	+	+	+	+
endo-1,4-beta-glucanase E1 precursor	cellulose	+	+	+	+	+
endo-1,4-beta-xylanase	xylan	+	+	+	+	+
endo-1,4-beta-xylanase Z precursor	xylan	+	+	+	+	+
alpha-glucosidase	glucoside	+	+	+	+	+
beta-glucosidase / beta-fucosidase	glucoside / fucoside	+	+	+	–	–
6-fosfo-beta-glucosidase / beta-galactosidase	glucoside / galactoside	+	+	+	–	+
malto-oligosyltrehalose trehalohydrolase	malto-oligosyltrehalose	+	+	+	–	–
4-carboxymuconolactone decarboxylase/alkylhydroperoxidase	lignin	+	+	+	–	+
catalase KatE	lignin	–	–	–	+	+
catalase-peroxidase KatG	lignin	+	+	+	–	–
catalase-like hemeprotein	lignin	+	+	+	+	+
manganese catalase	lignin	+	+	+	+	+
dye-decoloring peroxidase	lignin	+	+	+	–	–
Bcp-type thiol peroxidase	lignin	+	+	+	+	+
alpha-amylase / alpha-manosidase	starch	+	+	+	+	+

**Table 6:** Proteins encoded in the genomes of *Brasilonema* spp. strains presenting functional predictions previously described as involved in plant-infection processes.

**Protein**	**Function**	**UFV-E1**	**CENA114**	**UFV-OR1**	**SPC 951**	**UFV-L1**
glucosamine-6-phosphate deaminase	chitin degradation	+	+	+	+	+
N-acetylglucosamine-6-phosphate deacetylase	chitin degradation	+	+	+	+	+
N-acetylglucosamine kinase	chitin degradation	+	+	+	–	+
N-acetylglucosamine-related transporter NagX	chitin degradation	+	+	+	+	+
ClpP class periplasmic serine protease	protein degradation	+	+	+	+	+
serine protease	protein degradation	+	+	+	+	+
rhomboid family serine protease	protein degradation	+	+	+	+	+
serpin family serine protease inhibitor	protein degradation	+	+	+	+	+
anthranilate phosphoribosyltransferase	auxin biosynthesis	+	+	+	+	+
phosphoribosylanthranilate isomerase	auxin biosynthesis	+	+	+	+	+
aromatic L-amino acid decarboxylase	auxin biosynthesis	+	+	+	–	+
indole-3-pyruvate decarboxylase	auxin biosynthesis	+	+	+	–	–
tryptophan synthase –alpha chain	auxin biosynthesis	+	+	+	+	+
tryptophan synthase –beta chain	auxin biosynthesis	+	+	+	+	+
auxin efflux carrier protein	auxin biosynthesis	+	+	+	+	+
exopolysaccharide synthesis protein	exopolysaccharide biosynthesis	+	+	+	–	+
exopolysaccharide production protein ExoZ	exopolysaccharide biosynthesis	+	+	+	+	+
exopolysaccharide production protein ExoQ	exopolysaccharide biosynthesis	+	+	+	–	+
Hop protein	type III secretion system effector	+	+	+	+	+

It is noteworthy that both nearly complete *B. octagenarum* genomes presented regions with content showing divergent composition when compared to their average content, which is commonly found in horizontally-acquired sequences, while further evidences of horizontal transfer were obtained by predictions of other mobile genetic elements (Fig. 4A). However, none of the sequences similar to known virulence factors in the *Brasilonema* genomes were found within regions estimated as originating from horizontal transfer, and thus these genes are unlikely to be of xenologous origin.

**Table 7:** Virulence factors synthesized by phytopathogens with similarity to proteins encoded in *Brasilonema* genomes. An expanded version of this table is provided as Table S1.

**UniProt accession**	**Protein**	**Function**	**UFV-E1**	**CENA114**	**UFV-OR1**	**SPC 951**	**UFV-L1**
A0A0K0GHK1	SreR	two-component system regulation	+	+	+	+	+
A0A0K0GI30	DetR	two-component system cytoplasmic signaling	+	+	+	+	+
A0A0U1YU79	PilT	twitching motility	+	+	+	+	+
A4K9H6	PhoP	two-component system regulation	+	+	+	+	+
A4QUT2	CPXB	catalase-peroxidase	+	+	+	–	–
B0LFQ7	Bx-Prx	reproduction and pathogenicity	+	+	+	+	+
C5A846	PidR	two-component system regulation	+	+	+	+	+
C5A9K4	AroB	3-dehydroquinate synthase	+	+	+	–	+
D4HUY4	YhbH	ribosome-associated modulation protein	+	+	+	+	+
D4HX24	RpoS	regulation of stress and starvation response	–	–	–	+	+
D4HXR8	AcrD	resistance-nodulation-cell division transport	+	+	+	+	+
D4I307	ArgD	N-acetylornithine aminotransferase	+	+	–	–	+
F5HCK8	IPMDH	3-isopropylmalate dehydrogenase	+	+	+	+	+
G4ML75	MET6	methyltetrahydropteroyltriglutamate-homocysteine S-methyltransferase	+	+	+	–	–
G4MTK2	MoARG1	arginine biosynthesis	+	+	+	+	+
G4MXC5	MoPRX1	peroxiredoxin peroxidase	+	+	+	+	+
G4N4N6	MoSFA1	S-(hydroxymethyl)glutathione dehydrogenase	+	+	+	+	+
G4NCL5	MGG_00383	hypothetical protein	+	+	+	+	+
I1RSU2	PKS9/KSA1	polyketide synthase	+	+	+	+	–
P87025	THR1	reductase	–	–	–	+	+
Q12634	BUF/BUF1	undefined	+	+	+	+	+
Q2LK92	BcPIC5/BcFKBP12	rapamycin sensitivity	+	+	+	+	+
Q4UQD0	XC_3703	cyclic di-GMP effector	+	+	+	–	+
Q4UTV7	XC_2466	aspartate alpha-decarboxylase	+	+	+	+	+
Q4UUL4	XC_2203	nucleotide diphosphate kinase	+	+	+	+	+
Q58PW8	HsvA	hrp-associated systemic virulence	–	–	–	–	+
Q5H3K9	ColR	undefined	+	+	+	+	+
Q6RKH3	PKS7	polyketide synthase	+	+	+	–	+
Q6RKH4	PKS6	polyketide synthase	+	+	+	–	+
Q6RKH5	PKS5	polyketide synthase	+	+	+	–	–
Q7WTQ9	AcrB	multidrug efflux pump	+	+	+	+	+
Q8PM59	PstB	phosphate metabolism	+	+	+	+	+
Q8XSV8	FabG2	3-ketoacyl-acyl carrier protein reductase	+	+	+	+	+
Q8Y0J2	FabG1	3-ketoacyl-acyl carrier protein reductase	+	+	+	+	+
Q9C1T0	ARG1	argininosuccinate lyase	+	+	+	+	+
Q9UWF0	CAM	undefined	–	–	–	–	+

## Discussion

The initial growth of *B. octagenarum* on the surface of *E. urograndis* leaves apparently occurred as a relationship of epiphytism, as no detrimental effects to the host plant were initially observed. *B. octagenarum* colonization of *E. urograndis* later provided benefits to the host, which was evidenced by the higher production of root and stem dry mass in comparison to the control group (Table 2). The production and release of plant growth regulators by cyanobacteria has been confirmed in several studies with the identification of abscisic acid, auxins, cytokinins, ethylene and gibberelins (Singh et al., 2017), and stimulus to plant growth caused by cyanobacteria has been observed when these microorganisms are used as biofertilizers (Jhala, Panpatte & Vyas, 2017; Muñoz Rojas et al., 2018). Therefore, it is likely that colonization by cyanobacteria stimulated the growth of the host plants by the release of plant growth regulators by *B. octagenarum*.

On the other hand, the consequential decrease in the Chl *a*/*b* ratio in leaves resulting in the lighter green areas observed in Fig. 2 is most likely a direct response to the decrease in light incidence caused by sunlight blockage by the epiphyllic cyanobacterial colonies. Chl *a*/*b* ratio tends to decrease in response to shading, as previously observed in experiments with decreased light incidence in *Tetrastigma hemsleyanum* (Dai et al., 2009). This likely represents the early stages of leaf damage by the cyanobacterium and could lead to more severe consequences to the host plant in case of prolonged exposure. As described by Aguiar et al. (2008), in plants that remain for long periods under nursery conditions favorable to the development of microbial mats, extended cyanobacterial colonization starts to act as a stress factor compromising the source/drain balance of eucalyptus plants and could result in necrosis.

Overall, the *Brasilonema* protein sequences that presented similarity to sequences in PHI-base could perform roles in diverse molecular mechanisms such as biosynthesis and degradation, transport, signaling, regulation, starvation response, reproduction, motility and other processes, including currently unknown functions. Ten matches between the translated protein sequences from the *Brasilonema* genomes and proteins deposited in PHI-base (C5A9K4, D4I307, F5HCK8, G4ML75, G4MTK2, Q4UTV7, Q4UUL4, Q8XSV8, Q8Y0J2 and Q9C1T0) are likely to indicate enzymes with roles in primary metabolism (Cooley et al., 1999; Namiki et al., 2007; O’Connell et al., 2013; Karki & Ham, 2014; Ramos et al., 2014; Feng et al., 2015; Saint-Macary et al., 2015; Zhang et al., 2015a). Since mutants for the inactivation of the genes that encode their homologues in pathogens usually present auxotrophy, the presence of genes for proteins homologous to those factors in the *Brasilonema* genomes does not necessarily reflect any additional capacity of these strains to colonize plant surfaces. Additionally, a number of sequences in the *Brasilonema* genomes have presented similarity with polyketide synthases of unknown functions deposited in PHI-base (UniProt accession numbers I1RSU2, Q6RKH3, Q6RKH4 and Q6RKH5), but since the inactivation of the genes encoding these enzymes have not affected pathogenicity, it is unlikely that their homologues in *Brasilonema* genomes have roles in plant colonization. Four sequences from the UFV-E1 genome matched proteins that have been described as part of two-component systems involved in pathogenic processes (accessions A0A0K0GHK1, A0A0K0GI30, A4K9H6 and C5A846), but since two-component systems control behavior not only during pathogenicity, but also in cell communication and environmental adaptation (Zschiedrich, Keidel & Szurmant, 2016), these proteins might also have biological roles other than plant colonization.

The remaining protein sequences with similarity to PHI-base entries could have important roles for plant colonization. Five *Brasilonema* sequences have presented similarity with proteins involved in regulatory cascades for virulence (D4HUY4, D4HX24, Q4UQD0, Q58PW8 and Q5H3K9) (Oh, Kim & Beer, 2005; Subramoni et al., 2012; An et al., 2014; Ancona, Li & Zhao, 2014; Santander et al., 2014). Two hypothetical proteins from PHI-base (G4NCL5 and Q2LK92) presenting similarity to *Brasilonema* sequences were previously shown to have significant effects on plant pathogenicity, including severe symptoms like necrosis (Gioti et al., 2006; Jeon et al., 2007). Three copies of the twitching motility gene *pilT* were annotated in the UFV-E1 genome, important for spreading pathogen cells on the surface of the host (Weller-Stuart et al., 2017). Three translated protein sequences from the UFV-E1 genome have shown similarity with catalase-peroxidases and peroxiredoxins from PHI-base (accessions A4QUT2, B0LFQ7 and G4MXC5), enzymes with very important roles in the maintenance of redox homeostasis and the protection of pathogen cells from oxidative damage brought by hydrogen peroxide produced by host cells (Tanabe et al., 2011; Fu et al., 2014; Mir et al., 2015; Santander, Figàs-Segura & Biosca, 2018). Genes for other proteins potentially involved in detoxification have also been predicted, including multidrug efflux pumps (D4HXR8 and Q7WTQ9), which may confer resistance to phytoalexins (Burse, Weingart & Ullrich, 2004), and S-(hydroxymethyl)gluthatione dehydrogenase (G4N4N6), which is produced by phytopathogens as protection against damage from nitric oxide (Zhang et al., 2015b).

Although the identification of sequences similar to virulence factors in the *Brasilonema* genomes represents a potential for these cyanobacteria to cause damage to plants, their role within *Brasilonema* interactions with their hosts warrants further functional studies for the evaluation of the conditions under which these genes can be expressed. Nevertheless, except for one gene encoding a putative secreted protein that is likely a distant homolog of the Hop effector protein (Table 5), which is also found in other cyanobacteria with a presumed function of kinase (Zhang et al., 2016), the type III secretion system as well as its effectors appear to be completely absent in the assembled *Brasilonema* genomes. This is in agreement with observations for other cyanobacteria showing that they present the types I, II and IV secretion systems, but not the type III (Gonçalves et al., 2019). Since the transmission of this secretion system can occur either by vertical or horizontal transfer (Jackson et al., 2011), its absence in *Brasilonema* spp. together with the lack of evidence for horizontal transfer in the encoding of other molecules provides further evidence that leaf damage in this species emerged independently and not from the acquisition of genetic material from plant pathogens.

The role of type III secretion systems together with cell wall-degrading enzymes, serine proteases, phytohormones, siderophores and exopolysaccharides, among other molecules, has been increasingly recognized in plant pathogenesis (Lindeberg, 2012; Melotto & Kunkel, 2013). Most phytopathogens that affect plant productivity (including species of *Xanthomonas*, *Pseudomonas*, *Agrobacterium*, *Ralstonia* and *Erwinia*) are gram-negative, as are cyanobacteria, and one of the main mechanisms of virulence and pathogenicity of these phytopathogens is the type III secretion system, through which effector proteins are injected into host tissues, bringing about several functions that promote pathogenic processes (Sherif et al., 2015). Consequently, even if potential virulence factors, plant cell wall-degrading enzymes and other molecules were produced by the cyanobacteria in contact with their hosts, the absence of the type III secretion system in *Brasilonema* spp. is therefore a great obstacle for these microbes to actively infect plant cells.

## Conclusions

*Brasilonema octagenarum* UFV-E1 efficient growth and colonization of the phyllosphere was shown to cause lighter green areas on *Eucalyptus urograndis* leaves as a result of the decrease in the Chl *a*/*b* ratio. Sequences similar to known virulence factors from phytopathogens were found not only in the genome of *B. octagenarum* UFV-E1, originally isolated from damaged *E. urograndis* leaves, but also in the genome of *B. octagenarum* UFV-OR1, isolated from orchid leaves but not reported as causing damage to its host. However, none of these sequences were located within horizontally transferred regions, suggesting that horizontal gene transfer did not play a significant role in the emergence of the capacity for damaging leaves by *Brasilonema*. If these proteins also act as virulence factors, leaf damage by this species may be either restricted in host range or triggered by a more complex, uncommon set of factors making it a phenomenon of rare occurrence and thus not commonly observed. Similar sequences were also found in the genomes of *B. octagenarum* CENA114, obtained from a wet iron water pipe, *B. bromeliae* SPC 951 and *Brasilonema* sp. UFV-L1, isolated respectively from bromeliad and ligustrum leaves, which could suggest that the potential for leaf damage is not restricted to a single phyllosphere species, but also present in *Brasilonema* strains from other habitats and species.

Nevertheless, it is unlikely that these cyanobacteria could be capable of injecting any virulence factors and effectors into their plant hosts considering that the type III secretion system is the main vehicle of infection in known phytopathogenic bacteria but it is absent in this genus. Therefore, even if virulence factors could be expressed by the cyanobacteria in contact with plants, they appear to have no way of reaching the cytoplasm of their hosts since their genomes do not encode the structural proteins necessary for this process. This makes damage by *B. octagenarum* largely an indirect consequence of the growth of cyanobacterial mats on the leaf surface, causing the blockage of sunlight and hindering photosynthesis by the host, which could possibly progress into more severe consequences after extended colonization. It is therefore possible that other epiphyllic cyanobacteria, even those that do not produce any potential virulence factors, also cause similar damage if specific environmental conditions allow for their biomass to achieve significant volume. Consequently, even though epiphyllic cyanobacteria are traditionally thought of as being universally beneficial to their hosts, benefits to plants may come at a cost that may outbalance them when certain conditions that greatly favor cyanobacterial growth are met.

##  Supplemental Information

10.7717/peerj.9158/supp-1 Table S1 Virulence factors synthesized by phytopathogens with similarity to proteins encoded in Brasilonema genomes, including the effects of deleterious mutations on the pathogenicity of the microorganisms that produced them

10.7717/peerj.9158/supp-2 Table S2 Number of genes found in Brasilonema genomes classified in COG functional categories and occurrences of level 3 GO terms Hits for GO terms that are not found in bacterial genomes are also included, but more likely represent functions that are only related on higher levels.

10.7717/peerj.9158/supp-3 Supplemental Information 1 Raw data for in vivo experiments Raw data from which Tables 1 and 2 were produced.

10.7717/peerj.9158/supp-4 Supplemental Information 2 Brasilonema octagenarum UFV-E1 genome sequence

10.7717/peerj.9158/supp-5 Supplemental Information 3 Brasilonema octagenarum CENA114 genome sequence

10.7717/peerj.9158/supp-6 Supplemental Information 4 Brasilonema octagenarum UFV-OR1 genome sequence

10.7717/peerj.9158/supp-7 Supplemental Information 5 Brasilonema bromeliae SPC951 genome sequence

10.7717/peerj.9158/supp-8 Supplemental Information 6 Brasilonema sp. UFV-L1 genome sequence

## Additional Information and Declarations

### Data Availability

The following information was supplied regarding data availability: The sequences are available at GenBank: CP030118–CP030123, QMEA00000000, QMEB00000000, QMEC00000000.
